# Simplified Approaches for the Production of Monocyte-Derived Dendritic Cells and Study of Antigen Presentation in Bovine

**DOI:** 10.3389/fvets.2022.891893

**Published:** 2022-06-09

**Authors:** Patricia Cunha, Florence B. Gilbert, Jennifer Bodin, Lise Godry, Pierre Germon, Sebastien Holbert, Rodrigo Prado Martins

**Affiliations:** ISP, INRAE, Université de Tours, UMR1282, Nouzilly, France

**Keywords:** dendritic cells, antigen presentation, cell culture, serum-free media, bovine

## Abstract

Dendritic cells are sentinels of the immune system responsible for the initiation of adaptive immune mechanisms. In that respect, the study of these cells is essential for a full understanding of host response to infectious agents and vaccines. In ruminants, the large blood volume facilitates the isolation of abundant monocytes and their derivation to other antigen-presenting cells such as dendritic cells and macrophages. However, the available protocols for the production of bovine monocyte-derived dendritic cells (moDCs) rely mostly on time-consuming and costly techniques such as density gradient centrifugation and magnetic sorting of cells. In this study, we describe a simplified protocol for the production of bovine moDC using conventional and serum-free media. We also employ moDC produced by this approach to carry out a flow cytometry-based antigen presentation assay adapted to blood fresh or frozen cells. The experimental strategies described here might enable the setup of studies involving a large number of individuals, requiring a large number of dendritic cells, or relying on the utilization of cryopreserved blood cells. These simplified protocols might contribute to the elucidation of cell-mediated immune responses in bovine.

## Introduction

Dendritic cells (DCs) are sentinel leukocytes that play an essential role in the initiation and regulation of immune response. These cells can take up and transport antigens to lymphoid organs in order to stimulate T-cells, bridging the innate and adaptive arms of immunity. Since their discovery in the 1970s (1), DCs have been implicated in a myriad of processes that include the immune response against pathogens, cancer cells, and allografts, as well as the induction and maintenance of self-tolerance (2).

Dendritic cells are reported as the only professional antigen-presenting cells (APCs) capable of attracting and activating both the CD4^+^ and CD8^+^ naïve T cells. They are also considered as the most efficient APC, since the decreased proteolytic activity and acidity of their endocytic compartments reduce the antigen digestion rate and, consequently, increase the availability of partially processed peptides for loading on major histocompatibility complex (MHC) (3). The balance of T-cell immunity mechanisms relies greatly on the activity of DC subtypes found in sites of inflammation, such as monocyte-derived dendritic cells (moDCs) that act as inducers of Th1, Th2, and Th17 responses (4).

In the early 1990s, the observation that human monocytes *in vitro* cultured with granulocyte-macrophage colony-stimulating factor (GM-CSF) and interleukin-4 (IL-4) generate DCs (5) paved the way for the description of protocols allowing the production of large numbers of these cells. Indeed, the production of moDCs represents an attractive alternative to cumbersome and poorly reproducible methods involved in the isolation of DCs from tissues (6). For this reason, moDCs have been widely used to study DC biology in different species, including human (7, 8), swine (9), sheep (10), and bovine (11, 12).

Protocols for the generation of moDCs start with the isolation of monocytes. In early times, this step was mostly based on the separation of monocytes from peripheral blood mononuclear cells (PBMC) by adherence to plastic plates (5). However, the advent of antibody-conjugated microbeads has simplified the separation of monocytes by magnetic sorting and this strategy has become the most adopted for studies involving moDCs in many species. First, Liebana et al. (13) took advantage of antihuman CD14^+^ microbeads that recognize the bovine ortholog molecule to isolate monocytes for the production of bovine monocyte-derived macrophages (moMac) and more recently, Park et al. (11) and Guzman et al. (12) employed this technology for the production of bovine moDCs. Although this approach is sound, the cost of necessary material represents a handicap for laboratories from low-income countries, for studies addressing a high number of samples or for protocols requiring a high number of cells.

Time and cost-efficient methods enabling the production of high numbers of DCs are necessary for medium and high-throughput applications such as antigen screenings for vaccine development or immunogenicity *in-vitro* trials. Such methods might foster a better understanding of bovine immunology and the development of therapeutic and prophylactic strategies for this species. In this study, we set out to propose simplified protocols for the production of bovine moDC free of cell separation by density gradient centrifugation and/or magnetic sorting. Additionally, we developed an antigen presentation assay, suitable for fresh or frozen samples and adaptable to serum-free conditions, based on moDC-T-cell autologous co-cultures and flow cytometry analysis.

## Materials and Methods

### Blood Sampling and Ethics Statement

Peripheral blood samples were collected by jugular venipuncture from Holstein cows in lactation (*n* = 12) bred in the Unité Expérimentale de Physiologie Animale (UEPAO INRAE, France). No diseases (infectious, metabolic, or reproductive) were recorded within 30 days prior to sampling for all the selected animals. For antigen presentation assays, animals showing memory T-cell response against *Staphylococcus aureus* were selected. Blood samples were collected into 10 ml vacuum tubes containing ethylenediaminetetraacetic acid (EDTA) (Vacutainer K2-EDTA, BD) and kept at room temperature until processing (<1 h). Animal handling and blood sampling were conducted with the approval of Ethics Committee of Val de Loire (France, DGRIs agreement APAFIS#29498-2021020410061759 v2) in strict accordance with all the applicable provisions established by the European directive 2010/63/UE.

### Separation of Buffy Coat

Blood samples were centrifuged at 1,000 g for 30 min at 20°C with gentle deceleration. Buffy coat cells were collected, resuspended with ACK Lysing Buffer (Thermo Fisher Scientific), and incubated for 5 min at room temperature for the lysis of residual erythrocytes. Afterwards, the cells were washed twice with wash buffer [Dulbecco's phosphate-buffered saline (DPBS) without Ca^2+^ and Mg^2+^ supplemented with 2% (v/v) fetal bovine serum (FBS) and 2 mM EDTA], resuspended in FACS buffer (DPBS without Ca^2+^ and Mg^2+^ supplemented with 2% (v/v) normal goat serum and 2 mM EDTA), and counted using the LUNA FL Automated Cell Counter (Logos).

### Production of Monocyte-Derived Dendritic Cells

Simplified protocol 1—Buffy coat cells were resuspended in moDC medium (RPMI 10% FBS, 2 mM glutamine, 1 mM sodium pyruvate, 100 nM nonessential amino acids, and 50 μM β-mercaptoethanol) supplemented with premium grade human recombinant GM-CSF and IL-4 (20 ng/ml of each, Miltenyi Biotec) at a concentration of 3 × 10^6^ cells/ml and seeded in polystyrene tissue culture gas plasma-treated 6 well-plates (Falcon) (3 ml/well). After 12–14 h of incubation at 38.5°C with 5% CO_2_, non-adherent cells were discarded, adherent cells were gently washed twice with warm DPBS without Ca^2+^ and Mg^2+^, and 3 ml of warm moDC medium were added per well. At day 3 of culture, 50% of medium was replaced with fresh moDC medium containing 40 ng/ml of GM-CSF and IL-4. At day 6 of culture, non-adherent and adherent moDCs were harvested after treatment with 0.05% (m/v) trypsin-EDTA (Gibco).

Simplified protocol 2—Buffy coat cells were used for the separation of PBMC using density gradient centrifugation as previously described (14). PBMC was resuspended in moDC medium supplemented with 20 ng/ml of human recombinant GM-CSF and IL-4 (Miltenyi Biotec) at a concentration of 3 x 10^6^ cells/ml and treated as described in simplified protocol 1.

**Figure 1:**
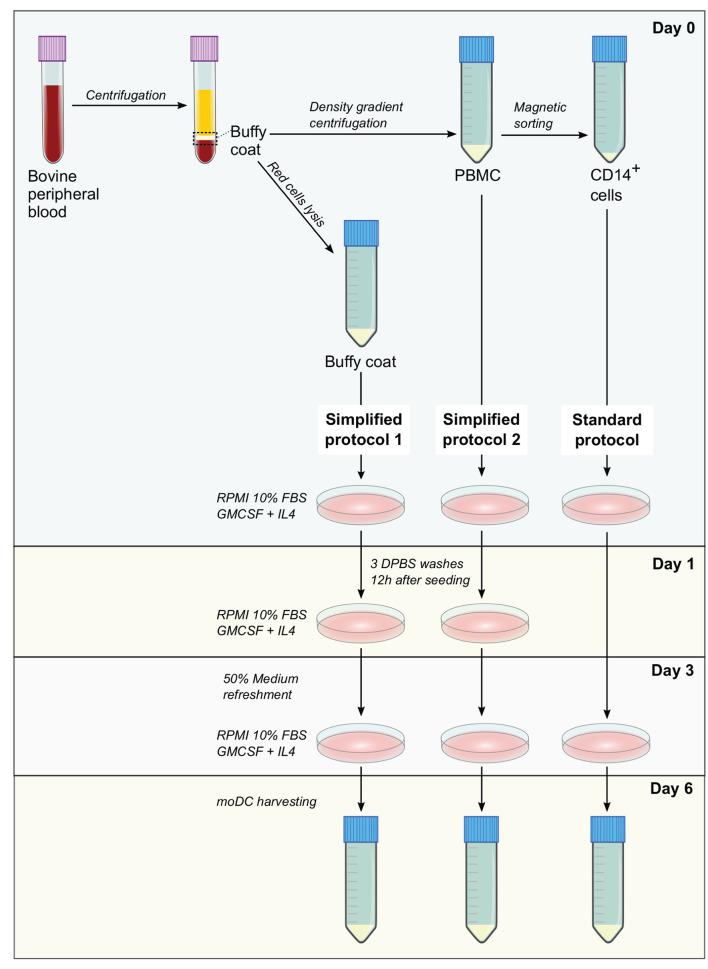
Methods for the production of bovine monocyte-derived dendritic cells (moDCs). In simplified protocols 1 and 2, buffy coat and peripheral blood mononuclear cells (PBMC) from ethylenediaminetetraacetic acid (EDTA)-treated whole blood are used as precursor cells for the derivation of moDCs, respectively. Non-adherent cells are washed out 12 h after seeding and after medium refreshment at day 3, moDCs are harvested at day 6. In standard protocol, moDCs are produced from CD14^+^ cells sorted from PBMC. After medium refreshment at day 3, moDCs are harvested in day 6.

Standard protocol—CD14^+^ cells were isolated from 3 × 10^7^ PBMC by magnetic sorting using CD14 microbeads (human, Miltenyi Biotec) according to manufacturer's conditions. Afterwards, 3 × 10^6^ cells were resuspended in 3 ml of moDC medium and seeded in tissue culture polystyrene gas plasma-treated 6 well-plates (Falcon). After 3 days, 50% of medium was replaced with moDC medium containing 40 ng/ml of GM-CSF and IL-4 and at day 6, non-adherent and adherent moDCs were harvested after treatment with 0.05% trypsin-EDTA (Gibco). A diagram illustrating the isolation of blood cells and the protocols for the production of moDC is given in Figure 1.

Serum-free media—Buffy coat cells were processed as described in simplified protocol 1 using AIM V Serum-free Medium (Gibco) supplemented with AlbuMAX (Gibco) or X-VIVO 15 serum-free hematopoietic cell medium (Lonza).

### Immunophenotyping by Flow Cytometry

Cells were harvested by treatment with 0.05% trypsin-EDTA or by gentle scraping in cold PBS containing 2 mM EDTA (for CD80 and CD86 labeling) and washed twice with FACS buffer. For the labeling of surface markers, the following mouse primary antibodies were used: anti-MHCII (CAT82A, IgG1, Kingfisher), anti-CD11c (BAQ153A, IgM, Biorad), anti-CD209 (209MD26A, IgG2a, Kingfisher), anti-CD11b (hybridome IAH CC104, IgG2b, kindly provided by Dirk Werling), anti-CD172a (CC149 conjugated to RPE-Cy5, Biorad), anti-CD14 (TUK4 conjugated to Alexa Fluor 647), anti-CD80 (IL-A159 conjugated to FITC), and anti-CD86 (IL-A190 conjugated to RPE). The following antimouse secondary antibodies were used when necessary: anti-IgG1 conjugated to Alexa 488 (A211121, Invitrogen), anti-IgM conjugated to PE-Cy7 (406513, Biolegend), anti-IgG2a conjugated to RPE (115-115-206, Jackson ImmunoResearch), and anti-IgG2b conjugated to BV510 (743175, BD Biosciences). After the antibody incubations, dead cells were labeled using the Fixable Viability Dye eFluor 450 (eBioscience). Cells were examined by flow cytometry using a BD LSR Fortessa cytometer and data were analyzed with the Kaluza software (Beckman Coulter). Dead cells were excluded from the analysis and gates were set according to appropriate isotype/control staining (Supplementary Figures 1, 3).

### Cell Stimulation With Lipopolysaccharide, Cytokines, and Bacteria

Monocyte-derived DCs harvested at day 6 were treated with LPS at 50 ng/ml (Sigma-Aldrich) or a cytokine cocktail composed of recombinant human IL-6 (Peprotech), recombinant human interleukin-1β (IL-1β) (Peprotech), and recombinant bovine interferon-γ (IFN-γ) (Biorad), each of them at 50 ng/ml, in moDC medium for 14 h at 38.5°C with 5% CO_2_. Treated cells were gently harvested with rubber scrapers in cold PBS with 2 mM EDTA. For stimulations with bacteria, moDC were harvested at day 9 and infected with a *S. aureus* strain (#1403) isolated from a bovine clinical mastitis at a multiplicity of infection (MOI) of 50 in moDC medium for 2 h under standard conditions. Afterwards, the cells were washed with DPBS and processed for reverse transcription-quantitative PCR (RT-qPCR) as follows.

### Reverse Transcription-Quantitative PCR

Cells were lysed in RA1 buffer supplemented with 1% (v/v) β-mercaptoethanol (Macherey-Nagel) and stored at −80°C until processing. Total RNA was extracted by using the NucleoSpin RNA Extraction Kit (Macherey-Nagel) and the residual genomic DNA was removed by DNase digestion with RNase-free DNase (Macherey-Nagel). The total RNA quantifications were carried out using a NanoDrop spectrophotometer (NanoDrop Technologies). For cDNA synthesis, total RNA (100 ng) was reverse transcribed using the 5X iScript reverse transcription supermix (Biorad) according to the manufacturer's instructions. cDNA samples were stored at −20°C until use. Reverse transcription-quantitative PCR assays were performed in a LightCycler 480 instrument (Roche). Four microliter of 10-fold diluted cDNA were added to a mixture of (2X) iTaq Universal SYBR Green Supermix (Biorad) and 0.25 μM of each primer in a total volume of 10 μl. Thermal protocol was 95°C for 5 min followed by 40 cycles of 95°C for 10 s, 60°C for 30 s, and acquisition of a melting curve at the end of the run. The specificity of primer pairs was checked via melting curve analysis. Primers used in this study (Supplementary Table 1) were designed and tested as previously described (14). Fold changes were calculated by the ΔΔCt method using ACTB, PPIA, and glyceraldehyde-3-phosphate dehydrogenase (GAPDH) as reference genes (15).

### Phagocytosis Assay

Monocyte-derived DCs harvested at day 6 were resuspended in moDC medium at a concentration of 2 x 10^6^ cells/ml and seeded in a tissue culture polystyrene vacuum gas plasma-treated 48 well-plates (Falcon) (100 μl/well). Cells were allowed to settle and adhere to the plate for 1 h at 38.5°C in a humidified incubator with 5% CO_2_. Afterwards, 100 μl of warm moDC medium supplemented or not with 1 μg of pHrodo Red *Escherichia coli* BioParticles Conjugate (Life Technologies) were added to treated and mock wells, respectively. After 1.5 h incubation at 38.5°C with 5% CO_2_, moDCs was collected with 0.05% trypsin-EDTA, washed twice with FACS buffer, and analyzed by flow cytometry. The phagocytic activity of cells was estimated based on the acidification of ingested particles, as the pHrodo dye increases in fluorescence in acidic pH conditions. The rate of phagocytosis was deduced from the percentage of cells showing high fluorescence emission at 590 nm, taking moDCs and pHrodo beads alone as reference.

### Antigen Presentation Assays Using Fresh Cells

Antigen pulsing—MoDCs harvested at day 6 were seeded in 48 well-plates (10^5^ cells/well in 500 μl of moDC medium) and incubated for 18 h at 38.5°C with 5% CO_2_. Then, cells were pulsed with a heat-inactivated (30 min at 70°C in PBS) *S. aureus* strain (#1403) isolated from a bovine clinical mastitis at a MOI of 50 for 2 h.

T-cell isolation and CellTrace labeling—At day 7, PBMC were isolated from autologous blood samples as described in the simplified protocol 2 and used for the separation of T cells by magnetic sorting. Briefly, 3 × 10^7^ PBMC were incubated with 3 μg of an anti-bovine CD3^+^ antibody (MM1A, Biorad) in 300 μl of FACS buffer for 30 min at 4°C. After 1 wash with FACS buffer, cells were resuspended in 240 μl of FACS buffer and 60 μl of antimouse immunoglobulin G (IgG) microbeads (Miltenyi Biotec), incubated for 30 min at 4°C, and washed with FACS buffer. CD3^+^ cells were sorted using MS columns (Miltenyi Biotec) and an octoMACS separator (Miltenyi Biotec) according to the manufacturer's conditions. T cells were subsequently labeled with 2.5 μM of CellTrace Violet (Invitrogen) using the manufacturer's protocol for cells in suspension and resuspended in X-VIVO medium at 5 × 10^5^ cells/ml.

**Figure 2:**
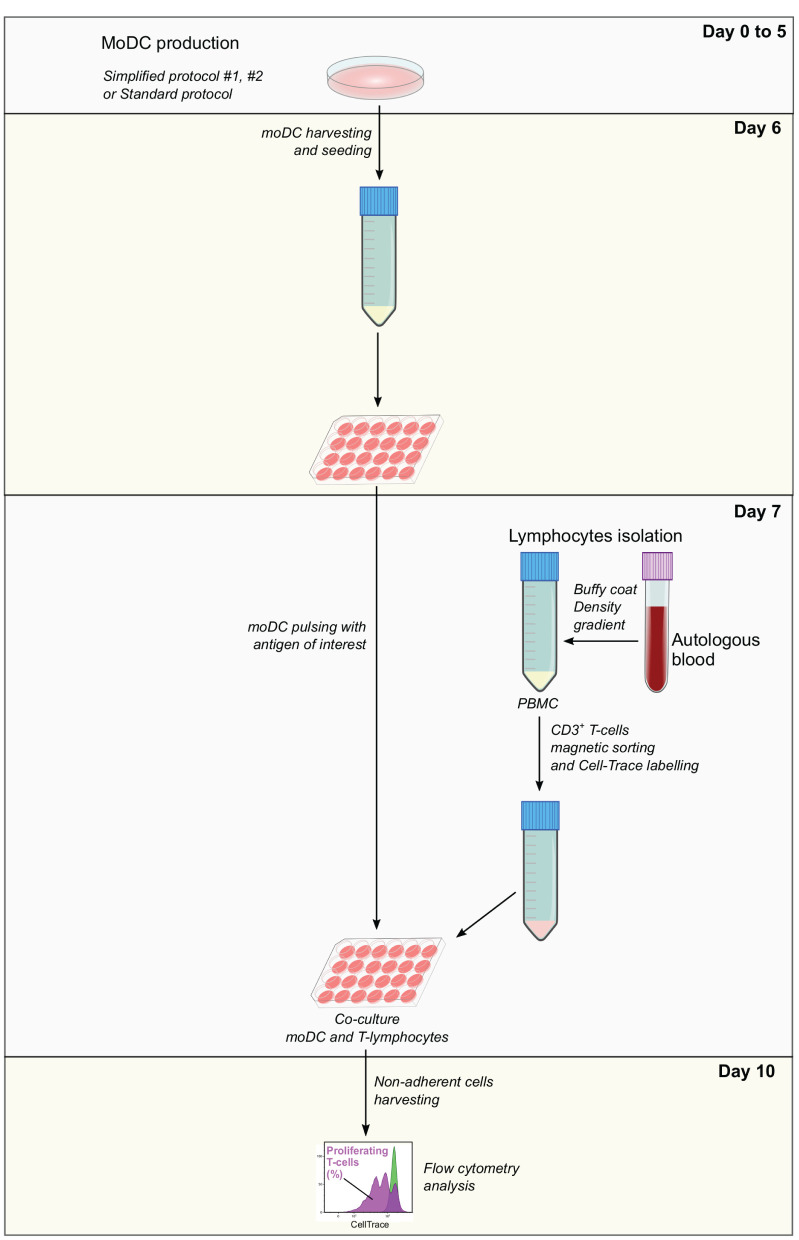
Antigen presentation assay. Monocyte-derived dendritic cells produced using simplified or standard protocols are harvested and pulsed with the antigen of interest at day 6. In parallel, autologous PBMC isolated from EDTA-treated blood samples are used to obtain CD3^+^ T cells by magnetic sorting. T cells are labeled with CellTrace Violet, co-cultured with pulsed moDC and after 3 days, non-adherent cells are collected and analyzed by flow cytometry. The levels of antigen presentation are estimated based on the percentage of proliferating T cells.

Co-culture and analysis by flow cytometry—After antigen pulsing, moDC medium was aspirated and replaced with 500 μl/well of the labeled T-cell suspension X-VIVO. After 3 days of co-culture (day 10), T cells were harvested from the supernatant and washed with FACS buffer before and after the staining of dead cells with the Fixable Viability Dye eFluor 660 (eBioscience). The percentage of T-lymphocytes proliferation was analyzed by flow cytometry based on the loss of CellTrace staining upon cell division using a BD LSR Fortessa cytometer. Data were analyzed with the Kaluza software (Beckman Coulter). Dead cells were excluded from the analysis and gates were set according to negative (lymphocytes alone) and positive (lymphocytes alone under polyclonal stimulation; 5 μg/ml of anti-bovine CD3 MM1A and 1 μg/ml of anti-bovine CD28 CC220 antibodies, Biorad) controls (Supplementary Figure 2). The levels of antigen presentation were estimated as a ratio between the percentage of T-cell proliferation upon co-culture with infected and control moDCs. A diagram illustrating each step of this assay is shown in Figure 2.

### Antigen Presentation Assays Using Frozen Blood Cells

**Figure 3:**
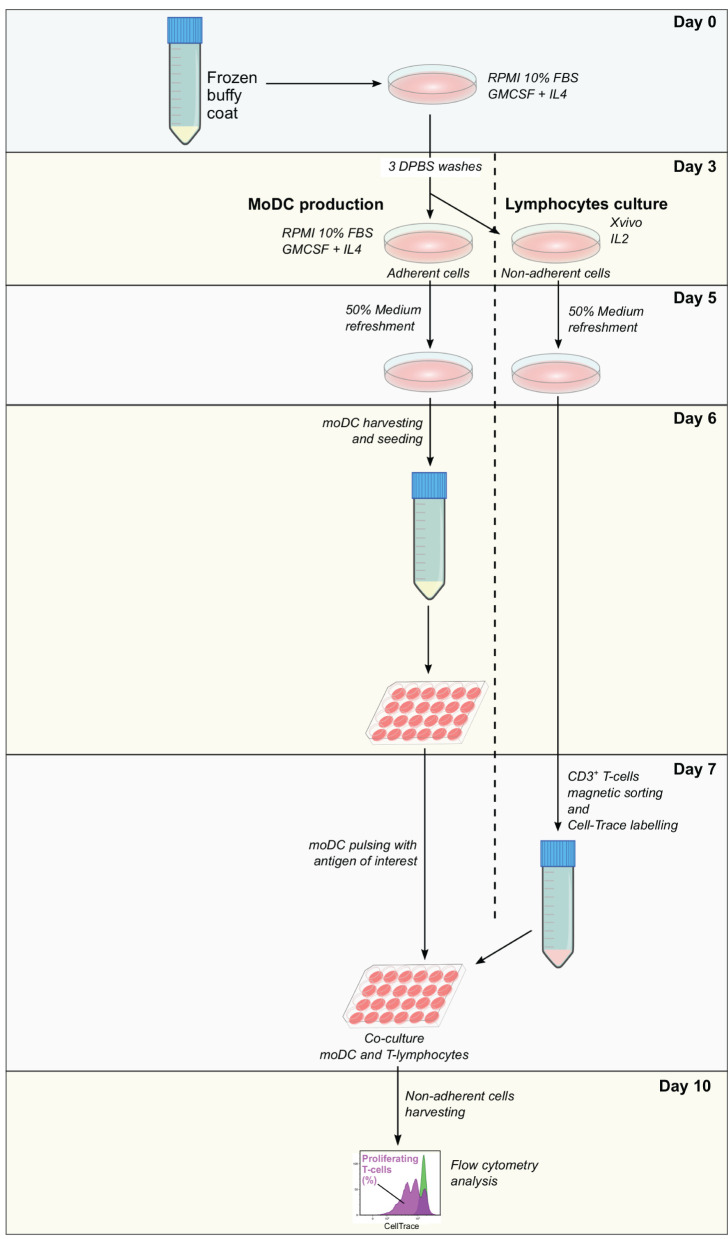
Antigen presentation assay using a single frozen sample. Frozen PBMC are thawed and incubated in RPMI 10% fetal bovine serum (FBS) supplemented with granulocyte-macrophage colony-stimulating factor (GM-CSF) and interleukin-4 (IL-4). After 3 days, non-adherent cells are removed and used for the expansion of lymphocytes, while adherent cells are used for the derivation of moDC according to the simplified protocol 1. At day 7, moDC is pulsed with the antigen of interest and CD3^+^ T cells are sorted from expanded lymphocytes. Pulsed moDC and CellTrace-labeled lymphocytes are co-cultured and after 3 days, non-adherent cells are collected and analyzed by flow cytometry. The levels of antigen presentation are estimated based on the percentage of proliferating T cells.

Frozen buffy coat cells were resuspended in moDC medium supplemented with 20 ng/ml of GM-CSF and IL-4 and seeded in tissue culture polystyrene gas plasma-treated 6 well-plates (10^7^ cells in 3 ml/well). After 3 days at 38.5°C with 5% CO_2_, non-adherent cells were collected, centrifuged, and resuspended in Xvivo medium supplemented with recombinant human IL-2 (Peprotech, 10 ng/ml) at 10^6^ cells/ml and seeded in 12 well-plates. Adherent cells were washed three times with warm DPBS and 3 ml of moDC medium supplemented with 20 ng/ml of GM-CSF and IL-4 were added per well. At day 5, 50% of medium from adherent and non-adherent cell cultures were replaced with fresh medium containing the doubled concentration of the corresponding cytokines. From day 6 to day 10, moDC seeding and pulsing, as well as T-cell isolation and labeling were performed as described in the protocol for fresh cells keeping the same proportion of cells and reagents, taking into consideration the cell yield. A diagram illustrating each step of this assay is given in Figure 3.

### Statistical Analysis

Data were analyzed and plotted using GraphPad Prism, version 6.0 (GraphPad Software Incorporation). The Kruskal–Wallis test was used to compare the independent groups and pairwise comparisons were carried out using the Dunn's test. Data shown represent the mean and the SD from at least three individual replicates (different cows).

## Results

### Immunophenotyping and Maturation of Monocyte-Derived Dendritic Cells

**Figure 4:**
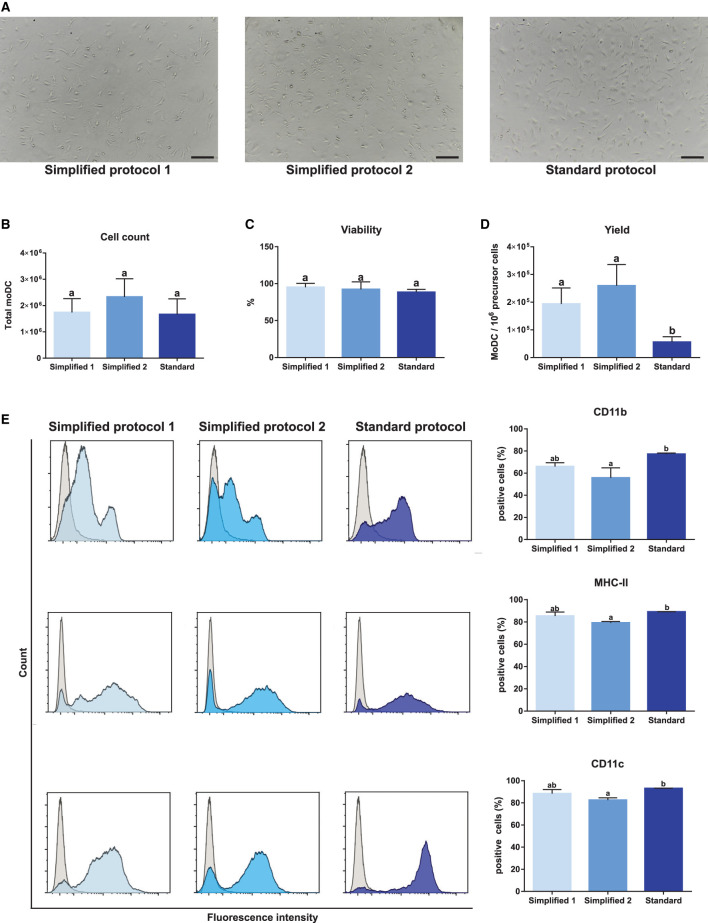
Yield, viability, and phenotype of bovine moDC produced by 3 different approaches at day 6. **(A)** moDC microscopic images, 100X magnification. Scale bars correspond to 20 μm. **(B)** Absolute moDC count. **(C)** Cell viability. **(D)** moDC count per 10^6^ of precursor cells. Buffy coat, PBMC, and CD14^+^ monocytes were used as precursor cells in simplified protocol 1, simplified protocol 2, and standard protocol, respectively. **(E)** Analysis of the surface markers CD11b, MHC-II, and CD11c by flow cytometry. The histograms (*left*) show the results of one representative experiment. Gray and blue peaks correspond to isotype and marker specific labeling, respectively. Bar plots (*right*) depict the percentage of positive cells. In **(B–E)**, bar plots depict data from individual replicates obtained from at least 3 and 4 different animals, respectively. Different letters mean statistically significant difference (*p* < 0.05). Error bars represent SD.

Monocyte-derived dendritic cells obtained by using two simplified methods were evaluated for their phenotype and maturation levels. A standard protocol based on magnetically-sorted CD14^+^ cells, as previously described for bovine samples (11), was used as a reference control. First, microscopic analyses showed no morphological differences among moDC produced using any of the three tested approaches (Figures 1, 4A). No significant differences were observed neither in the total number nor in the viability of the obtained cells (Figures 4B,C). However, as shown in Figure 4D, the simplified protocols 1 and 2 showed a higher yield when the number of derived moDC was estimated taking into consideration the number of precursor cells (buffy coat cells, PBMC, and sorted CD14^+^ cells, respectively). The analysis of moDC surface markers at day 6 by flow cytometry uncovered similar levels of CD11b-, MHC-II-, and CD11c-positive cells produced using both the simplified 1 and standard protocols. Nevertheless, a lower percentage of cells positive for these markers was observed when simplified 2 and standard protocols were compared (Figure 4E). Of note, intermediate levels of CD11b expression were found out for moDC produced by employing any of the simplified protocols (Figure 4E, upper histograms).

**Figure 5:**
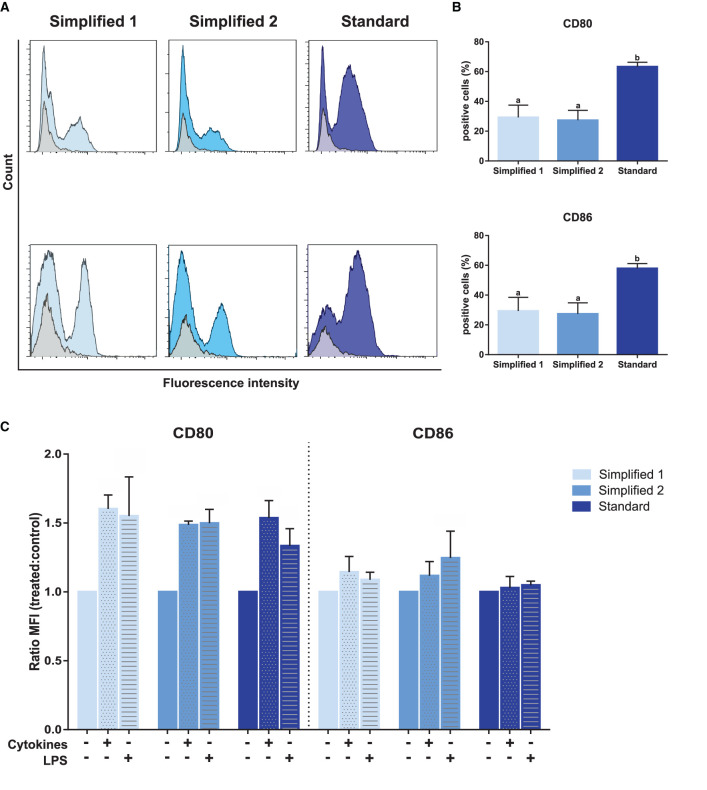
Analysis of maturation and dendritic cells-specific surface markers. **(A)** CD80 and CD86 detection by flow cytometry. Gray and blue peaks correspond to isotype and marker-specific labeling, respectively. The histograms show the results of one representative experiment. **(B)** Bar plots depict the percentage of positive cells in three independent samples. Different letters represent mean statistically significant difference (*p* < 0.05). **(C)** CD80 and CD86 MFI was analyzed after moDC treatment with a cocktail of cytokines [interleukin-6 (IL-6), interleukin-1β (IL-1β), and interferon-γ (IFN-γ)] or lipopolysaccharide (LPS). Bars corresponding to non-treated controls are identified as cytokines and LPS negative. Ratio values of samples from three different animals were calculated as follows: cytokine-treated MFI/mock MFI or LPS-treated MFI/mock MFI. Error bars represent SD.

The evaluation of CD80 and CD86 expression, two dendritic cells maturation markers, in immature moDCs at day 6, revealed a higher proportion of positive cells when the standard protocol was employed (Figures 5A,B). Then, the maturation of these cells was induced upon treatment with LPS or a cocktail of cytokines containing IL-6, IL-1β, and IFN-γ. As shown in Figure 5C, treatments induced an increase of CD80 levels in moDCs produced by employing any of the tested protocols. However, no increase of CD86 levels was observed for any of the tested groups upon cells stimulation.

**Figure 6:**
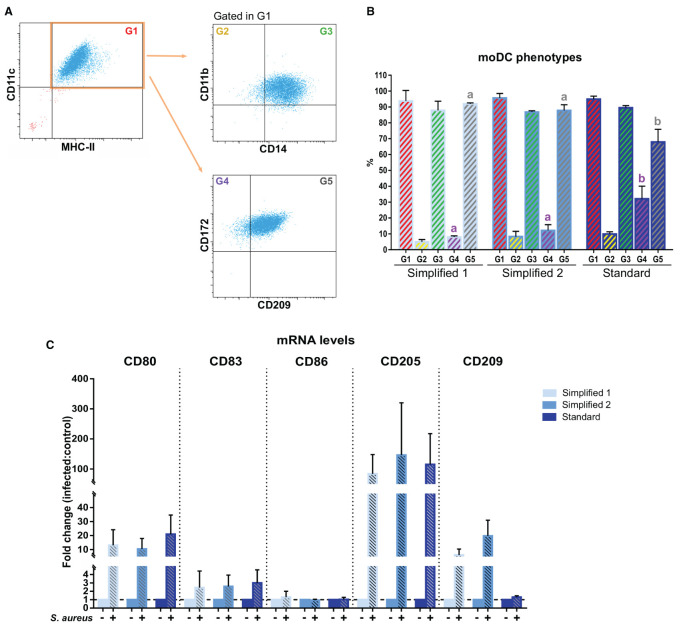
Phenotype of bovine moDCs produced by 3 different approaches at day 9. **(A)** Analysis of surface markers by flow cytometry. The dot plots show the results of one representative experiment. Monolabeled and isotype controls are shown in Supplementary Figure 1B. **(B)** Bar plot depicts the percentage of cells in the gates indicated in **(A)**. Bars represent data from three independent samples. Different letters represent mean statistically significant difference (*p* < 0.05). **(C)** CD80, CD83, CD86, CD205, and CD209 messenger RNA (mRNA) levels were analyzed after moDCs pulsing with a mastitis-causing *Staphylococcus aureus* (*S. aureus*) strain. Bars corresponding to non-treated controls are identified as *S. aureus* negative. Data from samples obtained from three different animals are shown as the fold change between the infected and control groups. Error bars represent SD.

We next complemented our analysis by evaluating the expression of CD14, CD209, and CD172a after 9 days of culture. In all the groups, about 90% of CD11c^+^ MHC-II^+^ cells co-expressed CD11b and CD14. Of note, the percentage of CD11c^+^ MHC-II^+^ cells co-expressing CD172a and CD209 was significantly lower in moDCs produced using the standard protocol (Figures 6A,B). Additionally, the maturation of moDCs upon an infection with *S. aureus* was verified by qPCR. As shown in Figure 6C, the exposure to bacteria induced the upregulation of CD80, CD83, CD86, and CD205 in all the moDC groups. Curiously, CD209 was less overexpressed in cells produced using the standard protocol.

### Phagocytic and Antigen Presentation Capacity of Monocyte-Derived Dendritic Cells

**Figure 7:**
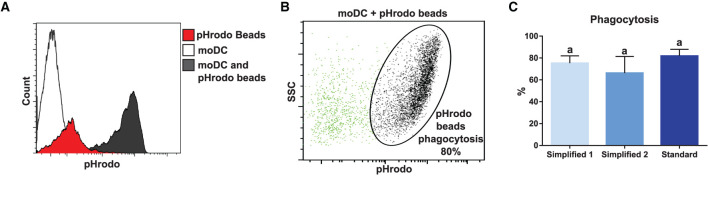
Analysis of phagocytosis capacity by flow cytometry. **(A)** White, red, and black peaks correspond to mock control, pHrodo beads alone, and moDC pulsed with pHrodo, respectively. Upon phagocytosis, pHrodo beads are exposed to acidic pH and have their fluorescence levels increased. The histogram shows the results of one representative experiment. **(B)** Dot plot shows non-phagocytic (green) and phagocytic (black) cells. The percentage of cells in the indicated gate corresponds to the percentage of phagocytosis. **(C)** The bar plot depicts the percentage of phagocytosis in samples from three different animals. Different letters mean statistically significant difference (*p* < 0.05). Error bars represent SD.

In order to compare the functional capacities of moDCs obtained by the tested approaches, we initially evaluated their capacity to uptake antigens. For this, we took advantage of the pHrodo-conjugated particles technology. Because pHrodo dye has its fluorescence increased under acidic conditions, the rate of phagocytosis was measured by flow cytometry upon the pulse of moDC with pHrodo Red *Escherichia coli* BioParticles Conjugate (Figures 7A,B). As shown in Figure 7C, moDCs produced by using any of the evaluated protocols proved to have a high antigen uptake capacity. Indeed, about 80% of cells were able to phagocyte particle conjugates after a 1.5-h exposure and no significant differences were observed between the tested groups.

**Figure 8:**
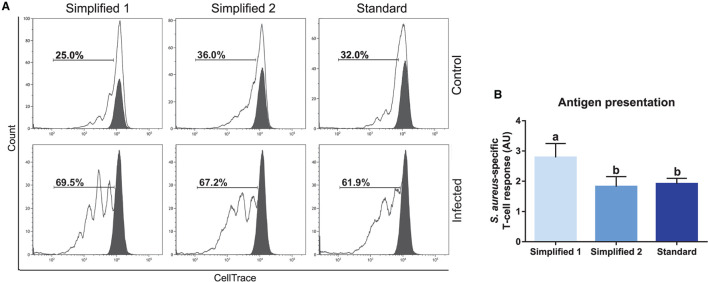
Antigen presentation assay. **(A)** The percentage of T lymphocytes proliferation was analyzed by flow cytometry after their co-culture with moDCs obtained by three different approaches. Black and white peaks correspond to lymphocytes alone (non-proliferating control) and exposed to moDCs, respectively. The gates indicate the percentage of proliferating lymphocytes. Control (upper) and infected (lower) lines correspond to lymphocytes co-cultured with infected or non-infected moDCs. Proliferation controls are shown in Supplementary Figure 2. Histograms show the results of one representative experiment. **(B)** The bar plot depicts the levels of *S. aureus-*specific T-cell response. Data were calculated as a ratio between the percentage of T-cell proliferation upon co-culture with infected and control moDCs. Samples from three different animals were used. Different letters represent mean statistically significant difference (*p* < 0.05). Error bars represent SD.

We also tested the capacity of moDC to present antigens by carrying out an assay given in Figure 2. Briefly, moDC was co-cultured with autologous T cells labeled to trace cell proliferation by flow cytometry. The percentage of T-cell proliferation in co-cultures with mock moDC and *S. aureus-*pulsed moDC was deduced based on the loss of cell trace staining upon cell divisions (Figure 8A). Then, the levels of antigen presentation were estimated as a ratio between the percentage of T-cell proliferation in co-cultures with infected and control moDC. As shown in Figure 8B, moDC produced using the simplified protocol 1 showed a better capacity to present bacterial antigens.

### Antigen Presentation Assay Using Frozen Peripheral Blood Mononuclear Cells

**Figure 9:**
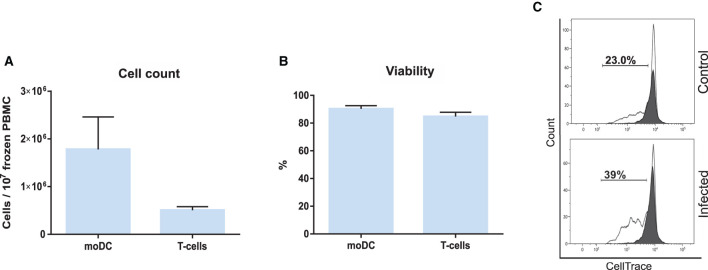
Antigen presentation assay using frozen PBMC. **(A)** moDC and T-cell count per 10^7^ frozen PBMC. **(B)** Cell viability. **(C)** The percentage of T lymphocytes proliferation was analyzed by flow cytometry. Control (upper) and infected (lower) graphs correspond to lymphocytes co-cultured with infected or non-infected moDC. Black and white peaks correspond to lymphocytes alone (non-proliferating control) and exposed to moDC, respectively. The gates indicate the percentage of proliferating lymphocytes. Histograms show the results of one representative sample out of three independent replicates from different animals. Error bars represent SD.

Based on the results of our initial screening, we next set out to adapt the simplified protocol 1 to study the antigen presentation using a single sample of frozen bovine blood cells. For this, adherent and non-adherent cells were separately kept in culture for the production of moDCs and isolation of CD3^+^ T cells (Figure 3). This approach enabled us to obtain from 10^7^ frozen buffy coat cells about 1.8 and 0.5 x 10^6^ moDC and T cells, respectively (Figure 9A). In both the cases, the viability levels observed were of about 90% (Figure 9B). These cells were used in the antigen presentation assay previously described and as shown in Figure 9C, the results obtained with frozen cells were comparable to the ones observed using fresh T cells. However, a higher variation of the *S. aureus-*specific T-cell response was observed using frozen cells (2.8 ± 1.9; mean ± SD; *n* = 3).

### Phenotypic and Functional Analysis of Monocyte-Derived Dendritic Cells Produced in Serum-Free Media

**Figure 10:**
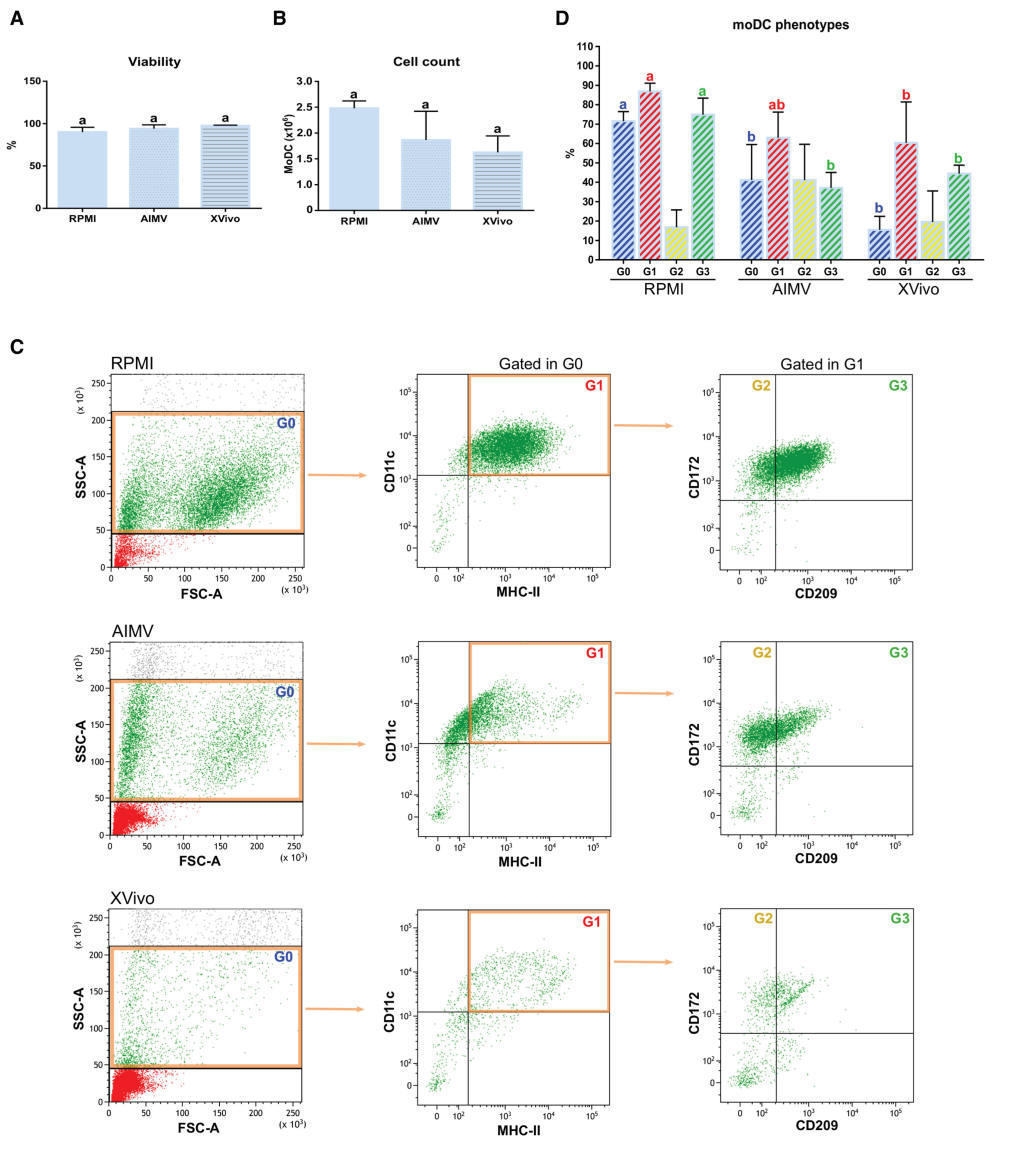
Viability, yield, and phenotype of bovine moDCs produced in RPMI-FBS and serum-free media (AIM V and X-VIVO) at day 9. **(A)** Cell viability. **(B)** Total cell count. **(C)** Analysis of the surface markers such as MHC-II, CD11c, CD172, and CD209 by flow cytometry. The dot plots show the results of one representative experiment. Monolabeled and isotype controls are shown in Supplementary Figure 3D. **(D)** Bar plot depicts the percentage of cells in the gates indicated in **(C)**. Bars represent data from three independent samples (from different animals). Different letters represent mean statistically significant difference (*p* < 0.05). Error bars represent SD.

Since the nonspecific activation of *in-vitro*-derived dendritic cells has been associated to the presence of FBS in the medium (16), we tested if the simplified protocol 1 could be used for the production of bovine moDCs under serum-free conditions. For this, cells from the same animals were processed using the serum-free media AIM V and X-VIVO 15 or RPMI supplemented with FBS (moDC medium) as a control. No differences were observed in the viability nor in the total number of cells obtained using any of the tested media (Figures 10A,B). Nevertheless, the phenotype of moDC produced in serum-free or FBS-supplemented media was different. As given in Figure 10C (left), less cells showing typical moDC size and internal complexity were observed in serum-free media. Additionally, moDC produced in absence of serum exhibited a lower expression of all the surface markers analyzed (Figure 10C middle and right). In fact, the percentage of cells co-expressing MHC-II, CD11c, CD172, and CD209 was significantly lower in both the serum-free media when compared to RPMI-FBS (Figure 10D).

**Figure 11:**
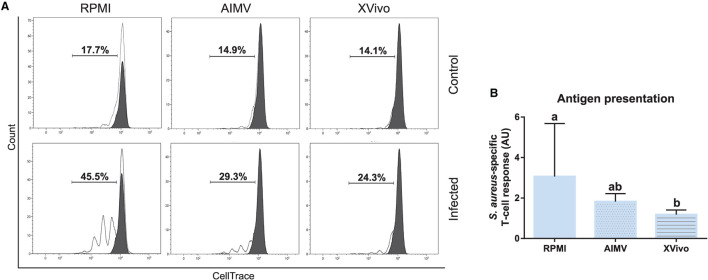
Antigen presentation assay with moDCs produced in RPMI-FBS and serum-free media (AIM V and X-VIVO). **(A)** The percentage of T-lymphocytes proliferation was analyzed by flow cytometry after their co-culture with moDCs produced in three different media. Black and white peaks correspond to lymphocytes alone (non-proliferating control) and exposed to moDCs, respectively. The gates indicate the percentage of proliferating lymphocytes. Histograms show the results of one representative experiment. **(B)** The bar plot depicts the levels of *S. aureus-*specific T-cell response. Data from three independent samples (from different animals) were calculated as a ratio between the percentage of T-cell proliferation upon co-culture with infected and control moDCs. Different letters represent mean statistically significant difference (*p* < 0.05). Error bars represent SD.

Subsequently, the functional activity of these cells was evaluated by using the antigen presentation assay given in Figure 2. As expected, CD3^+^ lymphocytes co-cultured with *S. aureus*-pulsed moDC proliferated at higher levels when compared to mock moDC, independently of the used medium (Figures 11A,B). However, higher rates of bacterial antigen presentation were observed in the FBS-supplemented medium.

## Discussion

Dendritic cells are major actors of host immune response in both the physiological and pathological contexts. In fact, the description of these cells and the development of approaches enabling their study represented a cornerstone for major discoveries in immunology (17). Unlike human DCs, which correspond to ~10% of steady-state blood leukocytes (6), DCs are hardly found in the blood of species such as mouse (18), swine (19), and bovine (20). Additionally, in spite of forming a vast network comprising peripheral and lymphoid organs (3), DC can only be isolated from tissues by using laborious and difficult to reproduce techniques (6). This fact represented a drawback for the DC biology field until the early 1990s, when culture systems based on the derivation of DCs from whole blood or bone marrow were described (5, 21).

Protocols for the production of bovine moDCs have been previously described (11, 12). However, they rely on the isolation of monocytes by magnetic sorting and their cost and yield can limit their application to medium- and large-scale studies, as well as by laboratories from low-income countries. To address this issue, we describe herein a simplified and cost-efficient protocol to produce large numbers of bovine moDCs from fresh or frozen cells in conventional or serum-free conditions.

We have tested two alternatives to the separation of monocytes with microbeads by using two different cellular starting materials. Upon the centrifugation of anticoagulant-treated bovine blood, the buffy coat is mostly composed of mononuclear cells, as granulocytes are found in the packed red cells fraction (22). We took advantage of this in the simplified protocol 1 by using cells from the buffy coat after the lysis of erythrocytes for the isolation of monocytes, circumventing density gradient centrifugations. This modification enabled us to simplify the first steps of the protocol and had no influence in the number or phenotype of the obtained cells, since no differences were revealed when cells derived by this approach were compared with those ones produced from PBMC obtained by density gradient centrifugation (simplified protocol 2). Our results also indicated that both the simplified protocols enabled the production of moDC with the same viability and similar phenotype when compared to the standard approach. Of note, the simplified protocols showed better yield and this difference could be associated to some modifications added to monocytes isolation step. We observed that an overnight incubation of buffy coat cells or PBMC with medium containing GM-CSF and IL-4 leads to a better monocytes adherence to plastic than the conditions previously described (2 h incubation without cytokines). Therefore, these modifications and a partial loss of monocytes when they are obtained by magnetic sorting could justify the better yield observed for the simplified protocols.

The multidimensional immunophenotyping of moDCs revealed that about 90% of cells MHC-II and CD11c positive co-expressed CD11b, CD14, CD172a, and CD209 when the simplified protocols were used. These results corroborate a previous analysis demonstrating that bovine moDCs expresses these surface markers (11, 23). However, some differences could be observed when the results of this study were compared with reports on bovine conventional DC. CD14 is a *bona fide* marker of the monocyte/macrophage lineage in bovine (24) that is absent in DCs from blood (20) and afferent lymph (12, 25). Therefore, the expression of CD14 by bovine moDCs corresponds to an unexpected result, but this observation has also been reported in moDCs from other domestic species (26, 27). In line with this study, CD209 is overexpressed in DCs from bovine blood (20), but no differences in the expression of this marker were found out when DCs and monocytes from bovine afferent lymph were compared (12).

Less homogeneous cell populations, marked by a higher proportion of cells negative for CD209, were observed when the standard protocol was used. In line with this observation, Guzman et al. (12) reported that the culture of magnetically-sorted bovine monocytes in the presence of GM-CSF, IL-4, and Flt3L generates heterogeneous cell populations.

The analysis of CD80 and CD86 expression, two markers of DC maturation, showed a higher number of cells carrying these molecules when the standard protocol was used. This result suggests that the isolation of monocytes by magnetic sorting leads to an early maturation of moDC. Besides this difference, LPS and a cocktail of cytokines induced moDC maturation independently of the method used for cell derivation. Curiously, moDC activation by the exposure to *S. aureus* induced the increase of CD80, CD205, and CD209 messenger RNA (mRNA), except for cells produced with the standard protocol, which failed to overexpress CD209. Changes in mRNA expression were not observed for CD83 and CD86, in line with a previous report showing that the infection with live *Mycobacterium avium subspecies paratuberculosis* does not affect CD80 or CD86 expression by bovine moDCs (23).

The functional analysis of moDCs revealed that cells obtained using the simplified protocols showed the same phagocytic activity as those ones derived with the standard approach. Cells generated with any of tested protocols could be used in the *in-vitro* antigen presentation assay described herein, but a better capacity to present bacterial antigens to T cells was uncovered for the simplified protocol 1. This observation and the ease of use of this protocol motivated us to adapt it for the production of moDC and study of antigen presentation using frozen cells and serum-free conditions. Frozen blood cells gave rise to moDC with a phenotype comparable to fresh cells (data not shown). Besides, we could obtain moDC and T cells from single frozen samples and use them in our antigen presentation test, producing results comparable to those ones observed with fresh cells. These observations indicate the feasibility of immunogenicity *in-vitro* tests using bovine blood cells in the context of field, large scale, or long-term studies relying on cryopreserved samples. Our simplified protocol was also suitable for serum-free conditions when AIM V, but not X-VIVO medium, was used. In serum-free conditions, moDCs showed CD11c and CD172a expression and antigen presentation capacity comparable to counterparts produced in FBS-supplemented medium. Nevertheless, contrary to a previous report (28), bovine moDCs produced in AIM V presented lower MHC-II expression than in RPMI-FBS medium.

When compared to a previous assay developed for immunogenicity studies in bovine (29), the experimental strategy described herein presents the following advantages: (i) it is based in a cost and time-efficient method for the production of moDCs; (ii) it is adaptable to serum-free conditions; (iii) it can be performed using frozen blood samples; and (iv) it estimates the levels of antigen presentation by a T-cell proliferation analysis, which does not rely on cell fixation and intracellular staining. This originality enables the use of our approach for generational analysis of T-cell proliferation in the context of antigen-specific responses, as well as for the sorting of live proliferating lymphocytes for clonal expansion, restimulation, and functional assays.

In conclusion, these simplified protocols might contribute to the elucidation of cell-mediated immune responses in bovine by enabling the setup of studies involving a large number of individuals, requiring a large number of cells, or relying on the utilization of cryopreserved blood cells.

## Data Availability Statement

The original contributions presented in the study are included in the article/Supplementary Material, further inquiries can be directed to the corresponding author.

## Ethics Statement

The animal study was reviewed and approved by Ethics Committee of Val de Loire (France, DGRI's agreement APAFIS#29498-2021020410061759 v2).

## Author Contributions

RPM: conceptualization. PC, FBG, JB, LG, and RPM: experimental work and data analysis. RPM, FBG, PG, and SH: resources. RPM, PC, SH, FBG, and PG: writing—original draft preparation and writing—review and editing. RPM and PC: supervision. RPM and SH: funding acquisition. All authors contributed to the article and approved the submitted version.

## Funding

This study was funded by the Agence Nationale de la Recherche (ANR-20-CE20-0023), INRAE Departement Santé Animale and INRAE UMR-ISP.

## Conflict of Interest

The authors declare that the research was conducted in the absence of any commercial or financial relationships that could be construed as a potential conflict of interest.

## Publisher's Note

All claims expressed in this article are solely those of the authors and do not necessarily represent those of their affiliated organizations, or those of the publisher, the editors and the reviewers. Any product that may be evaluated in this article, or claim that may be made by its manufacturer, is not guaranteed or endorsed by the publisher.
