# Supplementary material for: Simplified Approaches for the Production of Monocyte-Derived Dendritic Cells and Study of Antigen Presentation in Bovine
Source: Front Vet Sci. 2022 Jun 9;9:891893. doi: 10.3389/fvets.2022.891893 (PMC9223769; doi:10.3389/fvets.2022.891893)
Supplement: Supplementary file 1 [file Table_1.pdf]

Supplementary table 1. Gene-specific oligonucleotide primers used for RTqPCR

Gene Symbol	Oligonucleotides (5'-3')<br>F: forward ; R: reverse	Accession number (GenBank)	Reference
18s rRNA	F: CGGGGAGGTAGTGACGAAA	AF176811	Bougarn et al. 2011
	R: CCGCTCCCAAGATCCAACTA		
ACTB	F: ACGGGCAGGTCATCACCATC	BT030480	Bougarn et al. 2011
	R: AGCACCGTGTTGGCGTAGAG		
PPIA	F: TCCGGGATTTATGTGCCAGGG	BC105173	Bougarn et al. 2011
	R: GCTTGCCATCCAACCACTCAG		
CD80	F: ACTTCTGTTCAGGCATCACC	NM_001206439.1	This study
	R: AGATCCGAAGGCTTGTCAGTT		
CD83	F: GAAGGGCAGAGAAACCTGAC	BC112861	This study
	R: AGAGGTGACTGGGAGGAAAG		
CD86	F: TCTGACCTGATAGTGCTGGC	NM_001038017	This study
	R: ACATCCTCTGAGGTTCTGGGT		
CD205	F: GGGAACAAGTCACAGTGGGT	NM_001001158	This study
	R: CAGTTGCAGAATGGGCAAGC		
CD209	F: CACCCTCGACCACTACACAG	NM_001145756.1	This study
	R: TGAAGAAGCCCAGTGAGACG		
