# Supplementary material for: Simplified Approaches for the Production of Monocyte-Derived Dendritic Cells and Study of Antigen Presentation in Bovine
Source: Front Vet Sci. 2022 Jun 9;9:891893. doi: 10.3389/fvets.2022.891893 (PMC9223769; doi:10.3389/fvets.2022.891893)
Supplement: Supplementary file 2 [file Image_1.pdf]

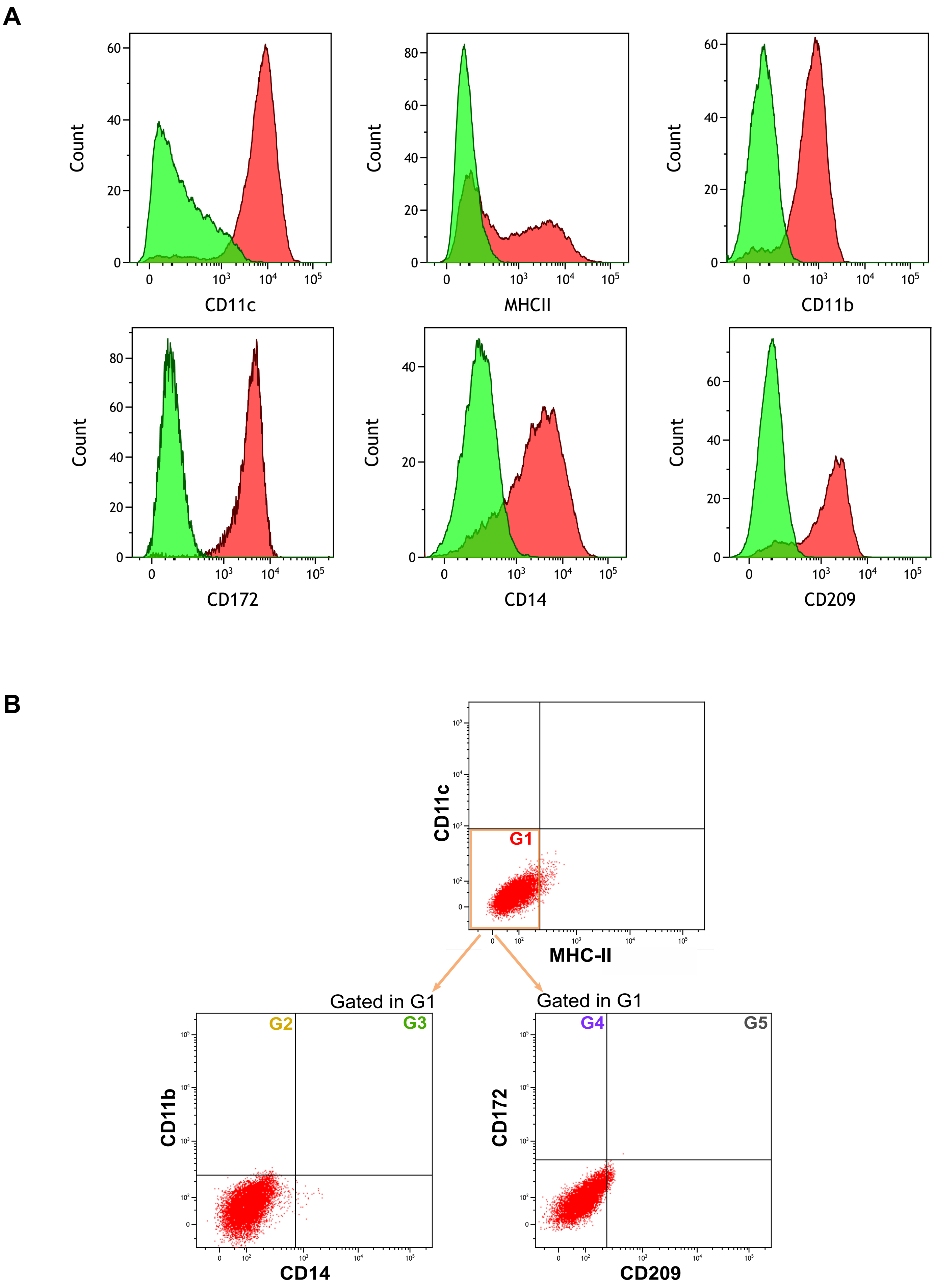

Supplementary Figure 1 - Controls for the flow cytometry-based analysis of dendritic cells surface markers shown in the Figure 6. **A:** Overlay histograms showing monolabelled (red) and isotype (green) controls. **B:** Gating strategy on non-stained cells (1:1:1 mix of moDC obtained with Simplified 1 and 2 as well as standard protocols).
