# Supplementary material for: Simplified Approaches for the Production of Monocyte-Derived Dendritic Cells and Study of Antigen Presentation in Bovine
Source: Front Vet Sci. 2022 Jun 9;9:891893. doi: 10.3389/fvets.2022.891893 (PMC9223769; doi:10.3389/fvets.2022.891893)
Supplement: Supplementary file 3 [file Image_2.pdf]

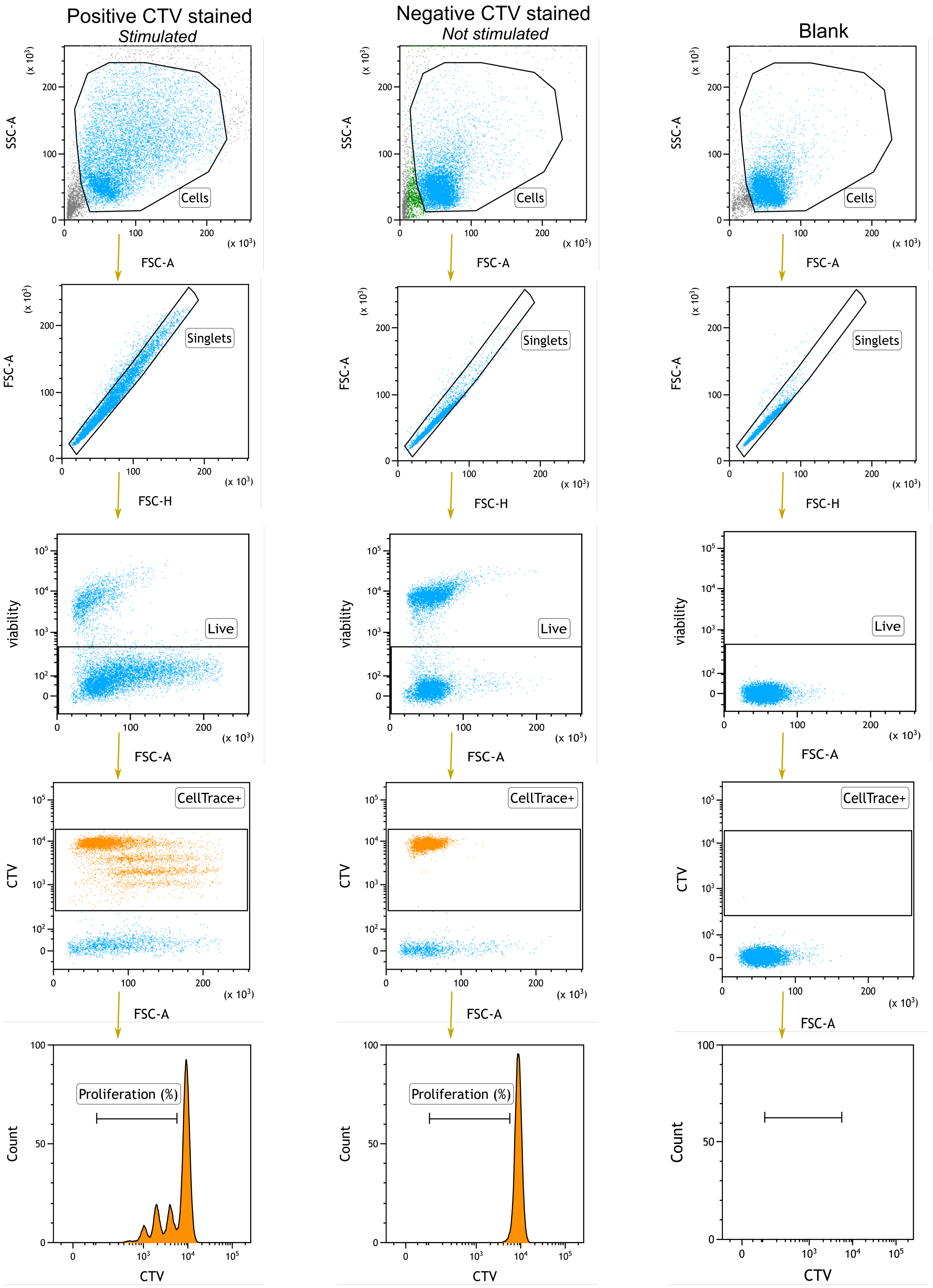

Supplementary Figure 2 - Gating strategy employed for antigen presentation data analysis by flow cytometry. Positive controls were treated with anti bovine CD3 and CD28 as described in methods. CTV = Cell Trace Violet.
